# Extracting Electron Densities in N-Type GaAs From Raman Spectra: Theory

**DOI:** 10.6028/jres.112.017

**Published:** 2007-08-01

**Authors:** Herbert S. Bennett

**Affiliations:** National Institute of Standards and Technology, Gaithersburg, MD 20899-8120

**Keywords:** complex dielectric response function, compound semiconductors, electron densities, line shape, non-destructive and contactless measurements, Raman spectra

## Abstract

In this paper, we present the theory for calculating Raman line shapes as functions of the Fermi energy and finite temperatures in zinc blende, n-type GaAs for donor densities between 10^16^ cm^−3^ and 10^19^ cm^−3^. Compared to other theories, this theory is unique in two respects: 1) the many-body effects are treated self-consistently and 2) the theory is valid at room temperature for arbitrary values of the ratio *R* = (*Q*^2^/*α*), where *Q* is the magnitude of the normalized wave vector and *α* is the normalized frequency used in the Raman measurements. These calculations solve the charge neutrality equation self-consistently for a two-band model of GaAs at 300 K that includes the effects of high carrier concentrations and dopant densities on the perturbed densities of states used to calculate the Fermi energy as a function of temperature. The results are then applied to obtain the carrier concentrations from Fermi energies in the context of line shapes in Raman spectra due to the coupling between longitudinal optical phonons and plasmons. Raman measurements have been proposed as a non-destructive method for wafer acceptance tests of carrier density in semiconductor epilayers. The interpretation of Raman spectra to determine the majority electron density in n-type semiconductors requires an interdisciplinary effort involving experiments, theory, and computer-based simulations and visualizations of the theoretical calculations.

## 1. Introduction

The carrier concentration is a key figure of merit associated with a go/no-go decision for determining whether a wafer or an epitaxial layer meets specifications and should undergo further processing. Technology roadmaps from the microelectronics and nanomaterials industries [1–3] call for non-destructive and fast-turn around methods to measure transport properties such as carrier concentrations in semiconductor wafers and epitaxial layers. Non-destructive measurements are economically more significant for III–V compound semiconductor wafers with epitaxial layers than for Si-based wafers with epitaxial layers because the regions of wafers used for making contacts cannot be used for product. Contacting measurement methods may be acceptable for Si wafers, but such destructive methods are much less acceptable for III–V compound semiconductor wafers with epitaxial layers because Si wafers are much less expensive per unit area than compound semiconductor wafers.

Raman spectroscopy has been proposed as one possible way to measure carrier concentrations non-destructively. The shape of the Raman spectral lines due to the longitudinal-optical phonons interacting with the plasmon collective modes of the electron gas (so called coupled longitudinal optical (LO) phonon-plasmon modes) provides information on transport properties of the electron gas in polar semiconductors [4–8]. The frequencies *ω* of the coupled modes are proportional to carrier concentrations, and the peak widths Δ*ω* of the coupled modes are proportional to the scattering rates due to electron-phonon interactions. The qualitative determination of carrier concentration and mobility, which is inversely proportional to the scattering rate, from Raman spectra is reasonably straightforward based on these proportionalities. The quantitative determination of carrier concentrations and mobilities requires more sophisticated modeling of the spectra. Many of these higher level models involve fitting the spectra with the Fermi energy as a parameter and then determining the carrier concentration from knowing the fitted Fermi energy [6].

Most interpretations of Raman measurements on compound semiconductors such as GaAs require physical models and associated input parameters that describe how carrier densities vary with dopant concentrations and Fermi energies. In this paper, we develop the theory for extracting electron densities from Raman measurements of n-type GaAs at room temperature. We introduce two main classes of models that relate carrier concentrations to the Fermi energy for a given temperature and donor dopant density:

- Bandgap narrowing (BGN) models based on two equivalent bands at the Γ point in the first Brilluoin zone, and
- Parabolic densities of states (PDOS) models with and without a quartic term in the electron energy dispersion *E*_cΓ_(***k***) for the Γ conduction sub-band, where ***k*** is the wave vector.

The BGN models include many-body quantum effects and bandgap narrowing. The many-body quantum effects describe the electron-electron, electron-hole, and dopant ion-carrier interactions. The results are unique in two respects: 1) the many-body effects are treated self-consistently and 2) the theory is valid at room temperature for arbitrary values of the ratio *R* = (*Q*^2^/*α*), where *Q* is the magnitude of the normalized wave vector and *α* is the normalized frequency used in the Raman measurements. Other reported work either is valid at low temperatures near 0 K for arbitrary ratios (*Q*^2^/*α*) [4] or is valid at arbitrary temperatures for ratios *R* typically *R* ≪ 1 [5–7].

## 2. Theory

Because the Fermi energy is one of the variables for calculating line shapes in the Raman spectra from semiconductors, we first present in Sec. 2.1 the theory for the BGN and PDOS models by which numerical methods give closed-form analytic expressions that relate carrier concentrations to Fermi energy *E*_F_, temperature *T*, and donor dopant densities *N*_D_. Then, within the context of these BGN and PDOS models, we develop in Sec. 2.2 the theory for calculating the line shapes observed in Raman spectra.

### 2.1 Fermi Energy and Electron Density

The temperature and donor dopant density are the independent input parameters for evaluating the Raman line shape factor given in Eq. (19). The Fermi energy depends in turn on the dopant density, in this case the donor density *N*_D_ and *T*. For given *N*_D_ and *T*, we present here models to compute the Fermi energy with full Fermi-Dirac statistics for the carriers at finite temperature. By using these models, we calculate the majority electron density as a function of the Fermi energy in zinc blende, n-type GaAs for donor densities between 10^16^ cm^−3^ and 10^19^ cm^−3^. These calculations solve the charge neutrality equation self-consistently in terms of two main classes of models: the bandgap narrowing (BGN) model and three different parabolic densities of states (PDOS) models.

Some researchers propose that Raman spectra may be a way to determine temperatures with spatial resolutions across wafers on the order of micrometers when the Fermi energy and dopant density are known from other independent measurements. Comparing the results given by the BGN and PDOS models, which are described below, also provides predictions on the sensitivity of the Fermi energy and Raman spectrum to variations in temperature.

#### 2.1.1 BGN Model

The bandgap narrowing (BGN) model is a two-band model with one equivalent conduction band and one equivalent valence band at the Γ point in Brilluoin space. The BGN model is related to earlier work on n-type GaAs [9] and includes modifications to the densities of states due to high concentrations of dopants, bandgap narrowing, and many-body effects associated with carrier-carrier interactions (carrier-carrier exchange and correlation). This BGN model is fully self-consistent and uses the Klauder self-energy (fifth level of approximation) to calculate the distorted-perturbed densities of states for the carriers. This BGN model has the following main features:

1. Many-body quantum effects of carrier-carrier interactions and carrier-dopant ion interactions, bandgap narrowing, and distorted-perturbed densities of states for the carriers [9],
2. Iterative and self-consistent solutions of the coupled charge neutrality equation and Klauder’s fifth level of approximation for the renormalized self-energy propagator from which the distorted-perturbed densities of states are calculated,
3. Full Fermi-Dirac statistics for the carriers at finite temperature, and
4. Statistical analyses to give closed-form analytic expressions from very large, calculated data sets for carrier densities as functions of the Fermi energy. Tables 1 and 2 contain the input parameters for the BGN calculations in Ref. [9] and for the BGN model given here.

The electron *n* and hole *h* concentrations in units of cm^−3^ at thermal equilibrium are given, respectively, by

$$n=\int_{-\infty }^{+\infty }f_{0}(E)\rho_{C}(E)dE\;\mathrm{and}\;h=\int_{-\infty }^{+\infty }\left[1-f_{0}(E)\right]\rho_{V}(E)dE,$$ (1)

where *ρ*_C_(*E*) and *ρ*_V_(*E*) are, respectively, the electron density of states for the equivalent conduction band and the hole density of states for the equivalent valence band [9], where *f*_0_(*E*) = {1 + exp[(*E* − *E*_F_)/*k*_B_*T*]}^−1^ is the Fermi-Dirac distribution function. The calculations incorporate the Thomas-Fermi expression for the screening radius,

$$r_{s}^{2}=-\frac{4\pi e^{2}}{\varepsilon \varepsilon_{0}}\int_{-\infty }^{+\infty }\frac{df_{0}(E)}{dE}\left[\rho_{C}(E)-\rho_{V}(E)\right]dE,$$ (2)

and the charge neutrality condition

$$N_{I}=n-h,$$ (3)

to compute self-consistently the Fermi energy *E*_F_ and the screening radius *r*_s_ for given values of the ionized dopant concentration *N*_I_ and temperature *T*. The static dielectric constant is *ε* and the permittivity of free space is *ε*_0_. The ionized dopant concentration is positive for n-type material (donor ions) and negative for p-type material (acceptor ions). The results reported here are for n-type material. For the case discussed here, *N*_I_ = *N*_D_. The results for the screening radius *r*_s_ are not reported here because they are not needed to extract carrier concentrations from Raman scattering measurements.

#### 2.1.2 PDOS Models

The PDOS models use parabolic densities of states for all equivalent bands and sub-bands. Unlike the BGN model in Ref. [9], PDOS models such as the four-band PDOS model for GaSb in Ref. [10] do not include modifications to the densities of states due to many-body effects and high concentrations of dopants and carriers because of computational limitations associated with treating a four-band model in the context of the Klauder self-energy method (fifth level of approximation).

The zero of energy is at the minimum energy value (bottom) of the conduction Γ sub-band, *E*_cΓ0_ = 0.0. The bottoms of the conduction L and X sub-bands are, respectively, at *E*_cL_ and *E*_cX_. The maximum energy value (top) of the degenerate valence Γ sub-band is −*E*_G_, where *E*_G_ is the intrinsic bandgap of GaAs. The split-off valence sub-band at Γ due to spin-orbit coupling is neglected. The probabilities for typical holes in equilibrium to occupy appreciably these states in the split-off valence sub-band at Γ are very low. This means that the Fermi energies should be sufficiently above the valence sub-band maximum at Γ. Placing exact limits on the Fermi energies for which the PDOS models are valid would be tenuous, because knowledge of how the various sub-bands move relative to one another due to the dopant concentrations considered here and due to many body effects is not adequate. Table 3 lists the input parameters for the PDOS models.

The heavy hole mass *m*_hh_ and light hole mass *m*_lh_ for the two degenerate sub-bands at the top of the valence band are combined to give an effective mass

$$m_{V\tilde{Α}}={\left(m_{\mathrm{hh}}{}^{3/2}+m_{\mathrm{lh}}{}^{3/2}\right)}^{2/3},$$ (4)

for the valence topmost sub-band, which becomes the equivalent valence band with a hole energy dispersion given by *E*_νΓ_(***k***) ≈ − *E*_G_ − (*ħ*^2^*k*^2^/2*m*_νΓ_*m*_0_).

The general expression for the parabolic densities of states for electrons and holes per band extrema and per spin direction is given by

$$\rho (E)=\frac{N_{e}4\pi V\sqrt{E}}{\left(8\pi^{3}\right){\left(\hbar^{2}/2m^{*}m_{0}\right)}^{3/2}},$$ (5)

where *N*_e_ is the number of equivalent ellipsoids in the first Brillouin zone, the volume of the unit cell is *V =a_L_^3^*, *a*_L_ is the lattice constant, m* is one of the effective masses listed in Tables 2 and 3 for the appropriate band extrema, and *m*_0_ is the free electron mass.

We sub-divide the PDOS models into the PDOS2, PDOS2NPG, and PDOS4 models. All three PDOS models include the equivalent valence band described by Eq. (4).

##### PDOS2 Model

The PDOS2 model uses one equivalent conduction band and one equivalent valence band at the Γ symmetry point in the Brilluoin space for the integrals that appear in Eqs. (1) to (3). The electron energy dispersion for the equivalent conduction band is *E*_cΓ_(***k***) ≈ *E*_cΓ0_ + (*ħ*^2^*k*^2^/2*m*_C_*m*_0_).

##### PDOS2NPG Model

The PDOS2NPG model is a two-band model with one equivalent conduction band and one equivalent valence band at the Γ point in Brilluoin space. It has no bandgap narrowing, but it includes the non-parabolicity for the electron energy dispersion in the equivalent conduction band at Γ. According to Ref. [11], we may include non-quadratic |***k***|*^l^* terms in the electron energy dispersion *E*_cΓ_(***k***) for the conduction Γ sub-band in GaAs when ***k*** is small, namely,

$$E_{c\Gamma }(k)\approx E_{c\Gamma 0}+\left(\hbar^{2}k^{2}/2m_{C}m_{0}\right)+\left(\xi /E_{G}\right){\left(\hbar^{2}k^{2}/2m_{C}m_{0}\right)}^{2},$$ (6)

where *ξ* is the non-parabolicity factor. We use the Kane three level ***k***·***p*** model [11], which does not include the conduction sub-bands at L and X, to include quartic terms in *E*(***k***) with *l* = 4.

##### PDOS4 Model

The PDOS4 model has three conduction sub-bands at the respective Γ, L, and X symmetry points in the Brilluoin space and one equivalent valence band at the Γ symmetry point. For the PDOS4, we modify here the PDOS model for GaSb in Ref. [10] so that it is valid for GaAs. It uses the parabolic electron energy dispersion *E*_cΓ_(***k***) for the conduction Γ sub-band in GaAs when ***k*** is small, namely, *E*_cΓ_(***k***) ≈ *E*_cΓ0_ + (*ħ*^2^*k*^2^/2*m*_C_*m*_0_). The general expression for the temperature dependence of conduction sub-band minima relative to the top of the valence band at Γ is given by [12],

$$E_{i}=E_{i0}-\left[A_{i}T^{2}/\left(T+B_{i}\right)\right]$$ (7)

in units of eV, where *i* = Γ, L, or X. The values for the coefficients *E_i_*_0_, *A_i_*, and *B_i_* are listed in Table 4. Because 8 permutations of the wave vector in the (111) direction exist, there are 8 L sub-band ellipsoids with centers located near the boundary of the first Brillouin zone. Also, because 6 permutations of the wave vector in the (100) direction exist, there are 6 X sub-band ellipsoids with centers located near the boundary of the first Brillouin zone. Since about half of each ellipsoid is in the neighboring zone, the number of equivalent sub-bands *N*_cL_ for the *E*_cL_ is four, and the number of equivalent sub-bands *N*_cX_ for the X sub-band is three.

In terms of the four-band PDOS4 model for room temperature n-type GaAs, the total density of states *ρ*_c_(*E*) for the majority carrier electrons in n-type GaAs then becomes

$$\rho_{c}(E)=\rho_{c\Gamma }(E)+\rho_{\mathrm{cL}}(E)+\rho_{\mathrm{cX}}(E),$$ (8)

where *ρ*_cΓ_(*E*), *ρ*_cL_(*E*), and *ρ*_cX_(*E*) are the sub-band densities of states for the conduction Γ, L, and X sub-bands with effective masses of *m*_cΓ_, *m*_cL_, and *m*_cX_, respectively. The density of states for the minority carrier holes is

$$\rho_{v}(E)=\rho_{v\Gamma }(E)$$ (9)

with an effective mass of *m_vΓ_*

### 2.2 Dielectric Response Function

The longitudinal optical (LO) phonons and plasmons interact in polar semiconductors such as GaAs to form LO phonon-plasmon modes. The theoretical line shape function *L*_A_(***q***,*ω*) of the Raman spectrum due to longitudinal optical (LO) phonon-plasmon coupled modes is then given for the configuration to which this theory will be applied by [8]

LA(q,ω)=(1−e−ℏω/kBT)−1(ω02−ω2ωTo2−ω2)Im{−1(q,ω)}, (10)

where ***q*** is the scattering wave vector, *ω* is the Raman angular frequency shift, *ħ* is the Planck constant, *k*_B_ is the Boltzmann constant, *T* is the temperature in Kelvin, *ω*_0_ = *ω*_TO_(1 + *C*_FH_)^1/2^ is a parameter with the dimensions of angular frequency, *C*_FH_ is the dimensionless Faust-Henry coefficient which includes the LO/transverse optical (TO) phonon scattering ratio, and *ω*_TO_ is the TO phonon angular frequency. Table 5 contains values for the parameters in Eq. (10). The total longitudinal dielectric response function *ε* (***q***,*ω*) in the random phase approximation (RPA) is described by

$$\varepsilon (q,\omega )=1+4\pi \chi_{\mathrm{VE}}+4\pi \chi_{L}(\omega )+4\pi \chi_{e}(q,\omega ),$$ (11)

where the dielectric susceptibility *χ*_VE_ is the contribution from valence electrons, *χ*_L_(*ω*) is the contribution from the polar lattice phonons, and *χ*_e_(***q***,*ω*) is the contribution from the conduction electrons. The high frequency dielectric constant *ε*_∞_ is defined to be *ε*_∞_ = 1 + 4*πχ*_VE_. Equation (11) then becomes for a binary semiconductor,

$$\varepsilon (q,\omega )=\varepsilon_{\infty }+4\pi \chi_{L}(\omega )+4\pi \chi_{e}(q,\omega ).$$ (12)

The contribution of the polar lattice is given by [8]

$$4\pi \chi_{L}(\omega )=\varepsilon_{\infty }\left(\frac{\omega_{\mathrm{LO}}^{2}-\omega_{\mathrm{TO}}^{2}}{\omega_{\mathrm{TO}}^{2}-\omega^{2}}\right),$$ (13)

when phonon damping may be neglected and where *ω*_LO_ is the LO phonon angular frequency.

Within the context of the RPA, the Lindhard expression [13] gives the electronic contribution to the dielectric response function 4*πχ_e_*^0^(***q***,*ω*) that describes light scattering by the conduction electrons in doped semiconductors. We introduce the collision relaxation time *τ* that describes the losses associated with electron-phonon and electron-dopant interactions and the corresponding angular collision frequency *Γ* = *τ*^−1^. Mermin [14] showed that replacing *ω* with *ω* + *iΓ* in the Lindhard expression for 4*πχ_e_*^0^(***q***,*ω*) fails to conserve the number of local electrons and therefore is not the correct way to include collision broadening in 4*πχ_e_*^0^(***q***,*ω*). Instead, Mermin assumed that within the framework of a relaxation time approximation the electron-phonon and electron-dopant interactions relax the electron density matrix to a local equilibrium density matrix [4,14]. He then obtained the following Lindhard-Mermin relation [14] for *χ*_e_(***q***,*ω*),

$$\chi_{e}(q,\omega )=\frac{\left(\omega +i\Gamma )\chi_{e}{}^{0}(q,0)\chi_{e}{}^{0}(q,\omega +i\Gamma \right)}{\omega \chi_{e}{}^{0}(q,0)+i\Gamma \chi_{e}{}^{0}(q,\omega +i\Gamma )},$$ (14)

where the Lindhard expression *χ_e_*^0^(***q***,*ω + iΓ*) for electrons occupying states in a single equivalent conduction band is given by,

$$\begin{array}{l} 4\pi \chi_{e}{}^{0}(q,\omega +i\Gamma )=\frac{e^{2}}{\pi^{2}q^{2}}\int f_{0}(E)\\ \times \left[{\left(\frac{\hbar^{2}q^{2}}{2m_{C}m_{0}}+\frac{\hbar^{2}q\cdot k}{m_{C}m_{0}}-\hbar (\omega +i\Gamma )\right)}^{-1}+{\left(\frac{\hbar^{2}q^{2}}{2m_{C}m_{0}}-\frac{\hbar^{2}q\cdot k}{m_{C}m_{0}}+\hbar (\omega +i\Gamma \right)}^{-1}\right]d^{3}k, \end{array}$$ (15)

where *f*_0_(*E*) = {1 + exp[(*E* − *E*_F_)/*k*_B_*T*]}^−1^ is the Fermi-Dirac distribution function, *E*_F_ is the Fermi energy in eV, and *T* is the temperature in Kelvin.

The integrand in Eq. (15) is independent of the azimuthal angle *φ* so that,

$$\int_{0}^{\infty }k^{2}dk\int_{0}^{\pi }\sin\theta d\theta \int_{0}^{2\pi }d\varphi (\ldots )=2\pi \int_{0}^{\infty }k^{2}dk\int_{-1}^{1}d\mu (\ldots )$$

where *µ* = cos*θ*, d*µ* = −sin*θ*, and ***q***·***k*** = *qkµ*. To simplify Eq. (15) further, we introduce the following dimensionless normalized quantities: *Q* = *qa*_B_, *K* = *ka*_B_, $K(E)=\sqrt{\left(2m_{0}m_{C}a_{B}{}^{2}/\hbar^{2}\right)E}$, and Ω^2^ = {*ħ*(*ω* + *iΓ*)*m*_C_/13.6 eV} where (*e*^2^/2*a_B_*) = (*ħ*^2^/2*m*_0_*a*_B_^2^) = 13.6 eV. Equation (15) then becomes with these definitions the following expression,

$$\begin{array}{l} 4\pi \chi_{e}{}^{0}(q,\omega +i\Gamma )\\ =\frac{4m_{C}}{\pi Q^{2}}\int_{0}^{\infty }K^{2}dK\\ \int_{-1}^{1}d\mu {\left(1+\exp\left[\left\{\left(13.6\mathrm{eV}K^{2}/m_{C}\right)-E_{F}\right\}/k_{B}T\right]\right)}^{-1}\\ \times \left[{\left(Q^{2}+2QK\mu -\Omega^{2}\right)}^{-1}+{\left(Q^{2}-2QK\mu +\Omega^{2}\right)}^{-1}\right]. \end{array}$$ (16)

Performing analytically the angular integration over *µ* gives the complex results

$$\begin{array}{l} 4\pi \chi_{e}{}^{0}(q,\omega +i\Gamma )\\ =\frac{2m_{C}}{\pi Q^{3}}\int_{0}^{\infty }KdK{\left(1+\exp[\{(13.6\;\mathrm{eV}\;K^{2}/m_{C})-E_{F}\}/k_{B}T]\right)}^{-1}\\ \times \left[\ln\left(\frac{\rho_{+}{}^{2}+\gamma^{2}}{\rho_{-}{}^{2}+\gamma^{2}}\right)+\ln\left(\frac{\tau_{+}{}^{2}+\gamma^{2}}{\tau_{-}{}^{2}+\gamma^{2}}\right)+i(\eta_{+}-\eta_{-})+i(\sigma_{+}-\sigma_{-})\right], \end{array}$$ (17)

where *ρ*_±_ = *Q*^2^ ± 2*QK* − *α*, *τ*_±_ = *Q*^2^ ± 2*QK* + *α*, *η*_±_ = −sign(*ρ*_±_)arctan(*γ*/*ρ*_±_) − (*π*/2)[1 − sign(*ρ*_±_)], *σ*_±_ = +sign(*τ*_±_)arctan(*γ*/*τ*_±_) + (*π*/2)[1 − sign(*τ*_±_)], Ω^2^ = *α* + *iγ*, *α* = (*ħωm*_C_/13.6 eV), *γ* = (*ħΓm*_C_/13.6 eV), and −*π* ≤ arctan(*ψ*) ≤ *π*.

When the temperature *T* = 0, the Fermi function *f*_0_(*E*) = (1 + exp[{(13.6 eV *K*^2^/*m*_C_) − *E_F_*}/*k*_B_*T*])^−1^ is the unit step function. Then, the integral in Eq. (17) $\int_{0}^{\infty }K^{2}dK(\ldots )$ is $\int_{0}^{K_{F}}K^{2}dK(\ldots )$, and an analytic evaluation is possible [4], where $K_{F}=\sqrt{m_{C}E_{F}/13.6\mathrm{eV}}$. But, when *T* is room temperature, analytic evaluations are not possible. Researchers approximated the integral $\int_{0}^{\infty }K^{2}dK(\ldots )$ at finite temperatures by expanding its integrand in terms of the ratio *R* = (*Q*^2^/*α*) for either very small or large *R*. However, such expansions do not capture all of the spectral information in Eq. (17).

The theoretical line shape function *L*_A_(***q***,*ω*) of the Raman spectrum then becomes

$$\begin{array}{l} L_{A}(q,\omega )\\ ={\left(1-e^{-\hbar \omega /k_{B}T}\right)}^{-1}\left(\frac{\omega_{0}^{2}-\omega^{2}}{\omega_{\mathrm{TO}}^{2}-\omega^{2}}\right)\left\{\frac{\varepsilon_{i}(q,\omega )}{\varepsilon_{r}{}^{2}(q,\omega )+\varepsilon_{i}{}^{2}(q,\omega )}\right\}, \end{array}$$ (18)

where Im{−1ε(q,ω)}={εi(q,ω)εr2(q,ω)+εi2(q,ω)}, εr(q,ω)=Re{ε(q,ω)}=ε∞{ωLO2−ωTO2ωTO2−ω2}+4πχre(q,ω+iΓ), and εi(q,ω)=Im{ε(q,ω)}=4πχie(q,ω+iΓ).

The Lindhard-Mermin relation Eq. (14) then gives expressions for *χ*_re_(***q***,*ω* + *iΓ*) = Re{*χ*_e_(***q***,*ω* + *iΓ*)} and *χ*_ie_(***q***,*ω* + *iΓ*) = Im{*χ*_e_(***q***,*ω* + *iΓ*)} as functions of *χ*_re_^0^ (***q***,*ω* + *iΓ*) = Re{*χ*_e_^0^(***q***,*ω* + *iΓ*)} and *χ*_ie_^0^ (***q***,*ω* + *iΓ*) = Im{*χ*_e_^0^(***q***,*ω* + *iΓ*)}. Even though the factors in Eqs. (10) and (18) give the contributions to the Raman line shape for each scattering mechanism, an examination of only the factor

Im{−1ε(q,ω)}={εi(q,ω)εr2(q,ω)+εi2(q,ω)}={(ωTO2−ω2)24πχie(q,ω+iΓ)Dr2(q,ω+iΓ)} (19)

gives the spectral information for extracting the Fermi energy. The denominator in Eq. (19) is given by the expression,

$$\begin{array}{l} D_{r}{}^{2}(q,\omega +i\Gamma )=\varepsilon_{\infty }{}^{2}{\left(\omega_{\mathrm{LO}}{}^{2}-\omega_{\mathrm{TO}}{}^{2}\right)}^{2}\\ +2\varepsilon_{\infty }\left(\omega_{\mathrm{LO}}{}^{2}-\omega^{2}\right)\left(\omega_{\mathrm{TO}}{}^{2}-\omega^{2}\right)4\pi \chi_{\mathrm{ie}}(q,\omega +i\Gamma )\\ +{\left(\omega_{\mathrm{TO}}{}^{2}-\omega^{2}\right)}^{2}\left[{\left\{4\pi \chi_{\mathrm{re}}(q,\omega +i\Gamma )\right\}}^{2}+{\left\{4\pi \chi_{\mathrm{ie}}(q,\omega +i\Gamma )\right\}}^{2}\right]. \end{array}$$ (20)

Section 3 below contains the procedures for extracting electron densities from the Fermi energies.

## 3. Numerical Results – Electron Density and Fermi Energy

Because the Fermi energy is one of the variables for calculating the Raman line shape, we give the numerical results for calculating the Fermi energy in terms of the BGN and PDOS models and the analytic expressions that relate the carrier concentrations to the Fermi energy.

### 3.1 BGN Model

We solve self-consistently, by means of an iterative procedure, Eq. (3) with the distorted-perturbed carrier densities of states *ρ*_C_(*E*) and *ρ*_V_(*E*) from Ref. [9] used in the numerical integrations for *n* and *h* given by Eq. (1). The *ρ*_C_(*E*) and *ρ*_V_(*E*) are in tabular form and have both localized and continuum states. Their associated band edges contain the many body effects related to exchange and correlation. The independent variables are the temperature *T* and donor density *N*_D_. The Fermi energy is varied for a given temperature until Eq. (3) is satisfied to within an error of plus or minus 10^−3^ × *N*_D_.

Numerical fitting procedures then give a closed-form analytic expression for the dependence of carrier concentration *n*_BGN_ on the Fermi energy *E*_F_; namely,

$$\begin{array}{l} \log_{10}\left(n_{\mathrm{BGN}}{\mathrm{cm}}^{3}\right)\\ =a_{\mathrm{BGN}0}+a_{\mathrm{BGN}1}E_{F}+a_{\mathrm{BGN}2}E_{F}^{2}+\ldots +a_{\mathrm{BGN}l}E_{F}^{l}\ldots . \end{array}$$ (21)

During the fitting analyses, we rely substantially on graphics and keep the number of fitting parameters to a minimum, subject to the constraint that the residual standard deviation *S*_res_ is acceptably small; i.e., *S*_res_ ≤ 0.01. The standard deviation is a measure of the “average” error by which a fitted model represents a set of data points and thereby is a metric for assessing the quality of the fit. A smaller *S*_res_ indicates a better fit. The residual standard deviation for a model *Y^f^* = *f* (*Z*) is

$$S_{\mathrm{res}}=\sqrt{\left[\sum_{j=1}^{N}{\left(Y_{j}-{\bar{Y}}_{j}^{f}\right)}^{2}/\left(N-P\right)\right]},$$ (22)

where *Y_j_* are the calculated data values, the ${\bar{Y}}_{j}^{f}$ are the predicted values from the fitted model, *N* is the total number of data points (here *N* = 28), and *P* is the total number of parameters to be fitted in the model. We use the NIST-developed DATAPLOT [15] software for both the exploratory graphics and for the statistical analyses.

In addition, we compare the above BGN model results with an equivalent two-band PDOS2 model for which there is no bandgap narrowing and for which the tabular carrier densities of states *ρ*_C_(*E*) and *ρ*_V_(*E*) from Ref. [9] are replaced with

$$\begin{array}{l} \rho_{C}\left(E\right)=\frac{N_{C}4\pi V\sqrt{E}}{\left(8\pi^{3}\right){\left(\hbar^{2}/2m_{C}m_{0}\right)}^{3/2}}\mathrm{and}\\ \rho_{V}\left(E\right)=\frac{N_{V}4\pi V\sqrt{E}}{\left(8\pi^{3}\right){\left(\hbar^{2}/2m_{V}m_{0}\right)}^{3/2}},\mathrm{respectively}. \end{array}$$ (23)

The corresponding polynomial fit for the carrier concentration *n*_0_ versus Fermi energy *E*_F_ relation is then denoted by

$$\log_{10}\left(n_{0}\;{\mathrm{cm}}^{3}\right)=a_{00}+a_{01}E_{F}+a_{02}E_{F}^{2}+\ldots +a_{0l}E_{F}^{l}\ldots .$$ (24)

**Figure 1:**
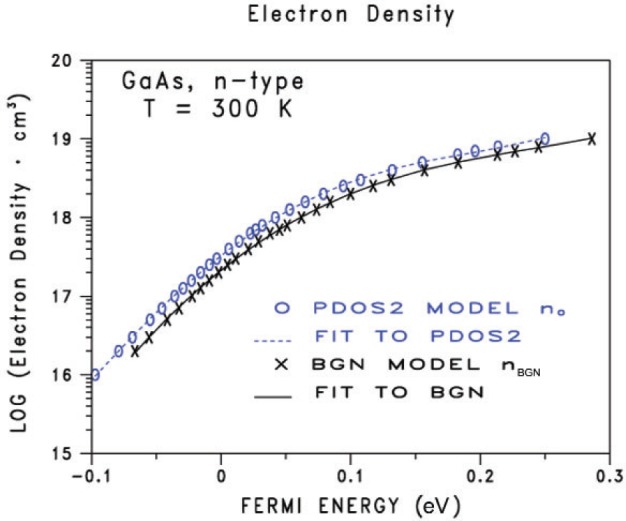
The calculated electron density *n*_BGN_ from the BGN model as a function of Fermi energy for n-type GaAs at 300 K is given by the solid-black curve. The calculated electron density *n*_0_ from the two-band PDOS2 model, no bandgap narrowing model, as a function of Fermi energy for n-type GaAs at 300 K is given by the dashed-blue curve. The Fermi energy is relative to the majority conduction band edge at the Γ symmetry point in the first Brillouin zone.

Figure 1 compares the calculated carrier concentration *n*_BGN_ and *n*_0_ data for 28 values of donor densities between 10^16^ cm^−3^ and 10^19^ cm^−3^. For a given Fermi energy, the electron densities predicted by the BGN model (solid-black curve) are typically factors of two smaller than the electron densities predicted by an equivalent parabolic two-band model (dashed-blue curve).

### 3.2 PDOS Models

We solve self-consistently, by means of an iterative procedure, Eq. (3) with Eq. (5). The independent variables are the temperature *T* and donor density *N*_D_.

We give here the results for fitting the logarithm to the base 10 of the total electron density and the electron densities in each of the three conduction sub-bands at Γ, L, and X, *n*_Γ_, *n*_L_, and *n*_X_, respectively, to polynomials in *E*_F_, namely,

$$\log_{10}\left(n_{t}\;{\mathrm{cm}}^{3}\right)=a_{t0}+a_{\mathrm{tl}}E_{F}+a_{t2}E_{F}^{2}+\ldots +a_{tl}E_{F}^{l}\ldots ,$$ (25)

$$\log_{10}\left(n_{\Gamma }\;{\mathrm{cm}}^{3}\right)=a_{\Gamma 0}+a_{\Gamma l}E_{F}+a_{\Gamma 2}E_{F}^{2}+\ldots +a_{\Gamma l}E_{F}^{l}\ldots ,$$ (26)

$$\log_{10}\left(n_{L}\;{\mathrm{cm}}^{3}\right)=a_{L0}+a_{\mathrm{Ll}}E_{F}+a_{L2}E_{F}^{2}+\ldots +a_{Ll}E_{F}^{l}\ldots ,\;\mathrm{and}$$ (27)

$$\log_{10}\left(n_{X}\;{\mathrm{cm}}^{3}\right)=a_{X0}+a_{\mathrm{Xl}}E_{F}+a_{X2}E_{F}^{2}+\ldots +a_{Xl}E_{F}^{l}\ldots .$$ (28)

**Figure 2:**
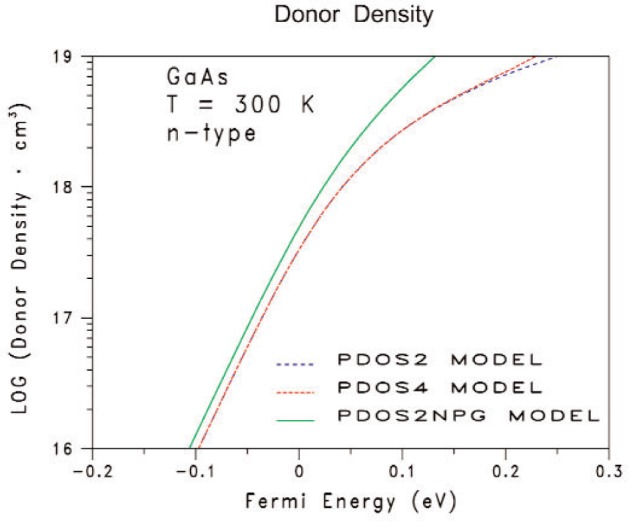
Comparisons among three PDOS models with and without the non-parabolic factor *ξ* in Eq. (5) for the electron energy dispersion *E*_cΓ_(*k*). The solid-green curve shows the results for an equivalent two-band model that has one equivalent Γ conduction band with non-parabolicity factor *ξ* and one equivalent Γ valence band; that is, the PDOS2NPG model. The dashed-blue curve with long spaces shows the results for an equivalent parabolic two-band model PDOS2 (*ξ* = 0). The dashed-red curve short spaces shows the results for the four-band PDOS4 model. The Fermi energy is relative to the majority conduction band edge at the Γ symmetry point in the first Brillouin zone.

**Figure 3:**
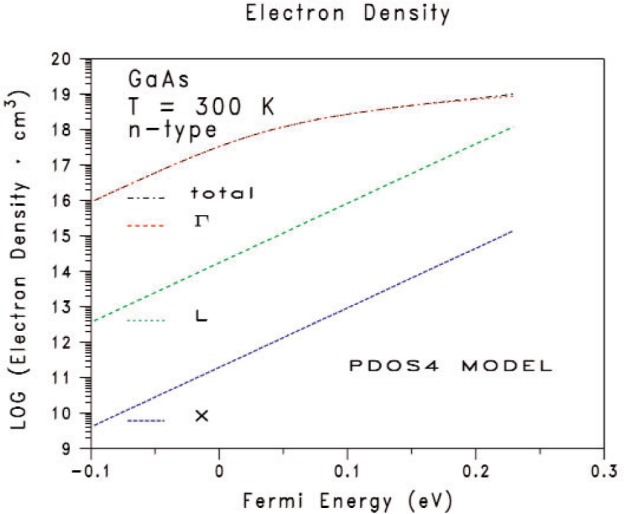
The calculated electron densities *n*_t_, *n*_Γ_, *n*_L_, and *n*_X_ from the four-band PDOS4 model as functions of the Fermi energy for n-type GaAs at 300 K. The Fermi energy is relative to the majority conduction band edge at the Γ symmetry point in the first Brillouin zone.

Figures 2 and 3 give the calculated electron densities as functions of the Fermi energy. The corresponding fitted curves are not shown in Figs. 2 and 3 because the pairs of curves (calculated and fitted) for each of the electron densities *n*_t_, *n*_Γ_, *n*_L_, and *n*_X_, lie on top of one another to within the line widths of each curve. Also, since the screening radii for the carriers from Eq. (2) are not needed when interpreting the proposed measurements considered here, the corresponding screening radii are not presented in this paper. Figure 2 compares the results from the PDOS2 model (dashed-blue curve with long spaces), PDOS2NPG model (solid-green curve), and PDOS4 model (dashed-red curve with short spaces). The data for the blue curve in Fig. 1 is the same data for the blue curve in Fig. 2. Figure 3 shows the results for the four-band PDOS4 model. Unlike GaSb, most of the electrons are in the conduction sub-band at *Γ*. The conduction sub-band at *L* is only weakly populated at the highest Fermi energies, and the conduction sub-band at *X* is negligible.

### 3.3 Polynomial Fits – Closed-Form Analytic Expressions

Tables 6 to 11 give the fitting parameters for polynomial fits to log_10_(*n*_BGN_ cm^3^), log_10_(*n*_0_ cm^3^), log_10_(*n*_t_ cm^3^), log_10_(*n*_Γ_ cm^3^), log_10_(*n*_L_ cm^3^), and log_10_(*n*_X_ cm^3^) as shown, respectively, in Eqs. (21) and (24) to (28) and the corresponding residual standard deviations *S*_res_. In general, the values of *S*_res_ decrease monotonically with increasing number *l* of terms in these polynomials. But, care must be taken to avoid fitting noise in data sets. The general guideline for many data sets is that when the *t*-ratio (i.e., the absolute value of the ratio of the estimated parameter value divided by its estimated standard deviation) is less than about 2, then the rate of decrease in *S*_res_ with increasing *l* tends to decrease, and proceeding with higher *l* values probably is not warranted. Because the changes in values of *S*_res_ between *l* = 4 and *l* = 5 are not experimentally significant, we use the fitting parameters for the quartic case *l* = 4 in Eqs. (21) and (24). Similary, because the changes in values of *S*_res_ between *l* = 3 and *l* = 4 are not experimentally significant, we use the fitting parameters for the cubic *l* = 3 case in Eqs. (25) to (28).

## 4. Conclusions

The foregoing theory for extracting electron densities from Raman spectra is unique in two ways: 1) It treats the many-body effects self-consistently, and 2) it is valid at room temperature for arbitrary values of the ratio *R* = (*Q*^2^/*α*). When high concentrations of carriers exists, this theory and its associated numerical procedures for determining carrier concentrations from Fermi energies are necessary for interpreting room temperature Raman spectra self-consistently for arbitrary values of the ratio *R*, even for *R* ≈ 1. The BGN models presented here include many-body quantum effects and bandgap narrowing. However, obtaining Fermi energies from experimental Raman spectra is beyond the scope of the present paper and involves the following steps to implement contactless measurements of carrier densities:

1. Numerical evaluation of Eq. (17) with adaptive grids to treat the integrable singularities of the integrands,
2. Three-dimensional visualization of computer results for predicted Raman line shapes as functions of Fermi energy and frequency, and
3. Iteration of the predicted Raman line shape with the Fermi energy as a variation parameter to give the best self-consistent fit to the measured Raman line shape.

## Figures and Tables

**Table 1:** Fundamental Constants

Parameter	Symbol	Value	Units
Planck’s constant	*ħ*	6.5836 × 10^−16^	eV·s
Boltzmann’s constant	*k*_B_	8.6174 × 10^−5^	eV/K
electron rest mass	*m*_0_	9.1072 × 10^−28^	g
electronic charge	*e*	−4.802 × 10^−10^	esu
Bohr radius	*a*_B_	0.5291 × 10^−8^	cm
energy associated with 1 Rydberg	*e*^2^/2*a*_B_	13.6	eV
speed of light	*c*	2.9979 × 10^10^	cm/s
wave length associated with 1 eV	[*λ*_0_]	1.2396 × 10^−4^	cm
wave number associated with 1 eV	[*k*_0_]	8.0668 × 10^3^	cm^−1^
dielectric constant in vacuum	*ε*	8.854 × 10^−12^	F/m

**Table 2:** BGN model input parameters for intrinsic zinc blende GaAs at 300 K. The energies of the extrema of the conduction and valence sub-bands are referenced to the bottom of the conduction sub-band at the Γ symmetry point in the Brillouin zone of the reciprocal lattice space. The mass of the free electron is *m*_0_. These GaAs data are from Ref. [12].

Parameter	Symbol	Value	Units
bandgap	*E*_G_ = |−*E*_vΓ_|	1.424	eV
effective mass for conduction band (2-band model) density of states	*m*_C_	0.067	*m*_0_
effective mass for valence band (2-band model) density of states	*m*_V_	0.572	*m*_0_
number of equivalent conduction bands	*N*_C_	1	
number of equivalent valence bands	*N*_V_	1	

**Table 3:** PDOS model input parameters for intrinsic zinc blende GaAs at 300 K. The energies of the extrema of the conduction and valence sub-bands are referenced to the bottom of the conduction sub-band at the *Γ* symmetry point in the Brillouin zone of the reciprocal lattice space. The mass of the free electron is *m*_0_. These GaAs data are from Refs. [11,12].

Parameter	Symbol	Value	Units
bandgap	*E*_G_ = |−*E*_vΓ_|	1.424	eV
bottom of the conduction L sub-band	*E*_cL_	0.29	eV
bottom of the conduction X sub-band	*E*_vX_	0.48	eV
top of the degenerate valence Γ sub-band	−*E*_vΓ_	1.424	eV
spin-orbit splitting	−*E*_so_	0.34	eV
top of the split-off (spin-orbit splitting) valence Γ sub-band	−*E*_soΓ_ = −*E*_vΓ_ − *E*_so_	1.764	eV
effective mass of conduction Γ sub-band	*m*_cΓ_	0.063	*m*_0_
non-parabolicity factor (quartic term prefactor) for conduction Γ sub-band	ξ	0.824	
transverse L sub-band mass	*m*_tL_	0.075	*m*_0_
longitudinal L sub-band mass	*m*_lL_	1.9	*m*_0_
effective mass of conduction L sub-band	*m*_cL_ = (*m*_lL_ *m*_tL_^2^)^1/3^	0.222	*m*_0_
transverse X sub-band mass	*m*_tX_	0.19	*m*_0_
longitudinal X sub-band mass	*m*_lX_	1.9	*m*_0_
effective mass of conduction X sub-band	*m*_cX_ = (*m*_lX_ *m*_tX_^2^)^1/3^	0.409	*m*_0_
light hole mass of valence Γ sub-band	*m*_lh_	0.082	*m*_0_
heavy hole mass of valence Γ sub-band	*m*_hh_	0.51	*m*_0_
effective mass of valence Γ sub-band	*m*_vΓ_	0.53	*m*_0_
splitoff band mass of the valence sub-band at Γ	*m*_so_	0.15	*m*_0_
number of equivalent conduction L sub-bands	*N*_cL_	4	
number of equivalent conduction X sub-bands	*N*_cX_	3	

**Table 4:** Coefficients for the temperature dependence of the conduction band extrema that are used in Eq. (15). These data are from Ref. [12].

Parameter	Symbol	Value	Units
Γ sub-band coefficients	*E*_Γ0_	1.519	eV
	*A*_Γ_	5.405 × 10^−4^	eV/K
	*B*_Γ_	204	K
L sub-band coefficients	*E*_L0_	1.815	eV
	*A*_L_		eV/K
	*B*_L_	204	K
X sub-band coefficients	*E*_X0_	1.981	eV
	*A*_X_	4.60 × 10^−4^	eV/K
	*B*_X_	204	K

**Table 5:** Dielectric response function input parameters for intrinsic zinc blende GaAs at 300 K. The energies of the extrema of the conduction and valence sub-bands are referenced to the bottom of the conduction sub-band at the Γ symmetry point in the Brillouin zone of the reciprocal lattice space. The mass of the free electron is *m*_0_. These GaAs data are from Ref. [14].

Parameter	Symbol	Value	Units
lattice constant	*a*_L_	5.65 ×10^−8^	cm
static dielectric constant	*ε*_0_	13.1	
high frequency dielectric constant	*ε*_∞_	10.9	
longitudinal optical (LO) phonon energy	*ħω*_LO_	0.0353	eV
		285	cm^−1^
transverse optical (TO) phonon energy	*ħω*_TO_	0.0332	eV
		268	cm^−1^
Energy associated with the angular collision frequency *Γ*	*ħΓ*	~0.0124	eV
due to electron-phonon and electron-dopant ion interactions		~100	cm^−1^
Faust-Henry coefficient	*C*_FH_	−0.4	
effective mass for the single equivalent conduction	*m*_C_	0.067	*m*_0_
band density of states in Eq. (6)			

**Table 6:** Bandgap narrowing BGN model for log_10_(*n*_BGN_ cm^3^). The five fitting parameters for a quartic polynomial fit Eq. (21) of the theoretical calculation for the equivalent conduction band electron density in n-type, zinc blende GaAs at 300 K as a function of the Fermi energy relative to the bottom of the equivalent conduction band at Γ. This quartic polynomial fit, which represents the theoretical results for Eq. (3), is valid only when −0.067 eV ≤ *E*_F_ ≤ 0.286 eV. The *t*-ratio is the absolute value of the estimated fitting parameter |*a*_BGN_*_i_*| divided by its estimated standard deviation. The residual standard deviation is *S*_res_ = 0.0130.

Fitting parameter	Estimated value	Estimated standard deviation	Units	*t*-ratio
*a*_BGN0_	17.3292	0.4191 × 10^−2^		4.135 × 10^3^
*a*_BGN1_	13.1545	0.7646 × 10^−1^	eV^−1^	1.72 × 10^2^
*a*_BGN2_	−37.4789	1.473	eV^−2^	25.45
*a*_BGN3_	26.5678	12.86	eV^−3^	2.067
*a*_BGN4_	53.7760	29.43	eV^−4^	1.827

**Table 7:** Two-band, no bandgap narrowing PDOS2 model for log_10_(*n* cm^30^). The five fitting parameters for a quartic polynomial fit Eq. (24) of the theoretical calculation for the L sub-band electron density in n-type, zinc blende GaAs at 300 K as a function of the Fermi energy relative to the bottom of the conduction Γ sub-band. This quartic polynomial fit, which represents the theoretical results for Eq. (3), is valid only when −0.0974 eV ≤ *E*_F_ ≤ 0.250 eV. The *t*-ratio is the absolute value of the estimated fitting parameter |*a*_0_*_i_*| divided by the its estimated standard deviation. The residual standard deviation is *S*_res_ = 0.0122.

Fitting parameter	Estimated value	Estimated standard deviation	Units	*t*-ratio
*a*_00_	17.5156	0.3411 × 10^−2^		5.136 × 10^3^
*a*_01_	12.8520	0.8063 × 10^−1^	eV^−1^	3.1 × 10^2^
*a*_02_	−34.5783	0.6672	eV^−2^	51.83
*a*_03_	−27.3728	9.166	eV^−3^	2.986
*a*_04_	223.720	29.54	eV^−4^	7.573

**Table 8:** Four-band PDOS4 model for total electron density log_10_(*n*_t_ cm^3^). The four fitting parameters for a cubic polynomial fit Eq. (25) of the theoretical calculation for the total electron density in n-type, zinc blende GaAs at 300 K as a function of the Fermi energy relative to the bottom of the conduction Γ sub-band. This cubic polynomial fit, which represents the theoretical results for Eq. (3), is valid only when −0.0974 eV ≤ *E*_F_ ≤ 0.229 eV. The *t*-ratio is the absolute value of the estimated fitting parameter |*a*_t_*_i_*| divided by its estimated standard deviation. The residual standard deviation is *S*_res_ = 0.0234.

Fitting parameter	Estimated value	Estimated standard deviation	Units	*t*-ratio
*a*_t0_	17.5198	0.6519 × 10^−2^		2.687 × 10^3^
*a*_t1_	12.3461	0.9401 × 10^−1^	eV^−1^	1.31 × 10^2^
*a*_t2_	−35.5542	1.322	eV^−2^	26.90
*a*_t3_	40.1630	6.488	eV^−3^	6.190

**Table 9:** Four-band PDOS4 model for electron density in the Γ sub-band log_10_(*n*_Γ_ cm^3^). The four fitting parameters for a cubic polynomial fit Eq. (26) of the theoretical calculation for the Γ sub-band electron density in n-type, zinc blende GaAs at 300 K as a function of the Fermi energy relative to the bottom of the conduction Γ sub-band. This cubic polynomial fit, which represents the theoretical results for Eq. (3), is valid only when −0.0974 eV ≤ *E*_F_ ≤ 0.229 eV. The *t*-ratio is the absolute value of the estimated fitting parameter |*a*_Γ_*_i_*| divided by its estimated standard deviation. The residual standard deviation is *S*_res_ = 0.0214.

Fitting parameter	Estimated value	Estimated standard deviation	Units	*t*-ratio
*a*_Γ0_	17.5187	0.5978 × 10^−2^		2.930 × 10^3^
*a*_Γ1_	12.3944	0.8621 × 10^−1^	eV^−1^	1.438 × 10^2^
*a*_Γ2_	−35.1795	1.212	eV^−2^	29.03
*a*_Γ3_	33.7647	5.950	eV^−3^	5.675

**Table 10:** Four-band PDOS4 model for electron density in the L sub-band log_10_(*n*_L_ cm^3^). The four fitting parameters for a cubic polynomial fit Eq. (27) of the theoretical calculation for the L sub-band electron density in n-type, zinc blende GaAs at 300 K as a function of the Fermi energy relative to the bottom of the conduction Γ sub-band. This cubic polynomial fit, which represents the theoretical results for Eq. (3), is valid only when −0.0974 eV ≤ *E*_F_ ≤ 0.229 eV. The *t*-ratio is the absolute value of the estimated fitting parameter |*a*_L_*_i_*| divided by its estimated standard deviation. The residual standard deviation is *S*_res_ = 0.001 17.

Fitting parameter	Estimated value	Estimated standard deviation	Units	*t*-ratio
*a*_L0_	14.2422	0.3269 × 10^−3^		4.357 × 10^4^
*a*_L1_	16.8180	0.4714 × 10^−2^	eV^−1^	3.568 × 10^3^
*a*_L2_	0.162418	0.6627 × 10^−1^	eV^−2^	2.451
*a*_L3_	−2.1988	0.3253	eV^−3^	6.759

**Table 11:** Four-band PDOS4 model for the electron density X sub-band log_10_(*n*_X_ cm^3^). The four fitting parameters for a cubic polynomial fit Eq. (28) of the theoretical calculation for the X sub-band electron density in n-type, zinc blende GaAs at 300 K as a function of the Fermi energy relative to the bottom of the conduction Γ sub-band. This cubic polynomial fit, which represents the theoretical results for Eq. (3), is valid only when −0.0974 eV ≤ *E*_F_ ≤ 0.229 eV. The *t*-ratio is the absolute value of the estimated fitting parameter |*a*_X_*_i_*| divided by its estimated standard deviation. The residual standard deviation is *S*_res_ = 0.000 019 5.

Fitting parameter	Estimated value	Estimated standard deviation	Units	*t*-ratio
*a*_X0_	11.2919	0.5452 × 10^−5^		2.071 × 10^6^
*a*_X1_	16.7999	0.7862 × 10^−4^	eV^−1^	2.137 × 10^5^
*a*_X2_	−0.542 16 × 10^−3^	0.1105 × 10^−2^	eV^−2^	0.4905
*a*_X3_	−0.4041 × 10^−2^	0.5426 × 10^−2^	eV^−3^	0.7448
